# Composites between Perovskite and Layered Co-Based Oxides for Modification of the Thermoelectric Efficiency

**DOI:** 10.3390/ma14227019

**Published:** 2021-11-19

**Authors:** Sonya Harizanova, Eric Faulques, Benoit Corraze, Christophe Payen, Marcin Zając, Dorota Wilgocka-Ślęzak, Józef Korecki, Genoveva Atanasova, Radostina Stoyanova

**Affiliations:** 1 Institute of General and Inorganic Chemistry, Bulgarian Academy of Sciences, 1113 Sofia, Bulgaria; sonya@svr.igic.bas.bg (S.H.); genoveva@svr.igic.bas.bg (G.A.); 2 Institut des Matériaux Jean Rouxel, University of Nantes, CNRS, IMN, F-44000 Nantes, France; Eric.Faulques@cnrs-imn.fr (E.F.); Benoit.Corraze@cnrs-imn.fr (B.C.); christophe.payen@cnrs-imn.fr (C.P.); 3 National Synchrotron Radiation Centre Solaris, 30-392 Kraków, Poland; mar.zajac@uj.edu.pl (M.Z.); jozef.korecki@ikifp.edu.pl (J.K.); 4 Jerzy Haber Institute of Catalysis and Surface Chemistry, Polish Academy of Sciences, 30-239 Kraków, Poland; dorota.wilgocka-slezak@ikifp.edu.pl

**Keywords:** cobalt-based perovskites, misfit layered oxides, thermoelectric oxides, multiphase composites

## Abstract

The common approach to modify the thermoelectric activity of oxides is based on the concept of selective metal substitution. Herein, we demonstrate an alternative approach based on the formation of multiphase composites, at which the individual components have distinctions in the electric and thermal conductivities. The proof-of-concept includes the formation of multiphase composites between well-defined thermoelectric Co-based oxides: Ni, Fe co-substituted perovskite, LaCo_0.8_Ni_0.1_Fe_0.1_O_3_ (LCO), and misfit layered Ca_3_Co_4_O_9_. The interfacial chemical and electrical properties of composites are probed with the means of SEM, PEEM/XAS, and XPS tools, as well as the magnetic susceptibility measurements. The thermoelectric power of the multiphase composites is evaluated by the dimensionless figure of merit, ZT, calculated from the independently measured electrical resistivity (ρ), Seebeck coefficient (S), and thermal conductivity (λ). It has been demonstrated that the magnitude’s electric and thermal conductivities depend more significantly on the composite interfaces than the Seebeck coefficient values. As a result, the highest thermoelectric activity is observed at the composite richer on the perovskite (i.e., ZT = 0.34 at 298 K).

## 1. Introduction

Nowadays, strongly correlated Co-based oxides are considered to be stable and less toxic thermoelectric alternatives to the conventional semiconductors, based on bismuth and tellurium [1]. Among these oxides, LaCoO_3_ with a perovskite structure and layered misfit Ca_3_Co_4_O_9,_ are the most interesting materials [2,3]. The LaCoO_3_ perovskite displayed a high Seebeck coefficient (|S| > 500 μV/K at room temperature), but low electric conductivity (about 10 Ωcm at room temperature) [4]. As a result, the thermoelectric activity was relatively low at room temperature (the dimensionless figure of merit *ZT* < 0.01 at T = 300 K). To improve the thermoelectric activity of LaCoO_3_, the metal substitution for the La- and Co-sites has mainly been explored [5]. This approach comprised of the replacement of the La^3+^ ions with alkaline earth metals or rare-earth elements, the replacement of Co^3+^ ions with 3d- or 4d- transition metals, as well as the creation of vacancies in both La- and O-sites [6,7]. Recently, we have demonstrated that nickel and iron co-substitution for Co ions in LaCoO_3_ led to a dramatic improvement of its thermoelectric efficiency [8]. This is a result from the reduction of the thermal conductivity through introducing specific structural disorders on the 6*b* perovskite site with multicomponent substitution for cobalt, while maintaining a high electric conductivity by preserving the perovskite-type structure as a whole [8,9]. Although the nickel ions led to a decrease in the Seebeck coefficient at the expense of the increase of electrical conductivity, the iron ions acted in an opposite way by increasing the Seebeck coefficient and decreasing the electrical conductivity. Through balancing the opposite effects of nickel and iron ions, the thermoelectric efficiency for double substituted perovskites, LaCo_0.8_Ni_0.1_Fe_0.1_O_3_, was improved in comparison with the single substituted analogues, LaCo_1-x_Ni_x_O_3_ and LaCo_1−x_Fe_x_O_3_ [8]. Contrary to Ni and Fe substituents, the replacement of La^3+^ with aliovalent Sr^2+^ ions had no effect on the thermal conductivity, and as a result of which the figure of merit was not improved for La_1-x_Sr_x_Co_0.8_Ni_0.1_Fe_0.1_O_3_ [9].

As in the case of perovskite oxides, the charge and spin-state of the Co ions in the misfit layered oxides, Ca_3_Co_4_O_9_, contributed mainly to the transport properties [10]. It has been recognized that the layer Ca_2_CoO_3_ was an insulating subsystem which maintains the charge supply for the conducting CoO_2_ layer, where both Co^3+^ and Co^4+^ ions coexist [11]. The transport properties could be easily modified by changing the separating block layer, as well as by alteration of the ratio between Co^3+^ and Co^4+^ ions. When the amount of Co^4+^ decreased, a significant increase in the Seebeck coefficient was achieved. The doping in CoO_2_-layer layers would influence the physical properties of the Ca_3_Co_4_O_9_ system more significantly [11].

To modify of the thermoelectric activity of both perovskites and layered oxides, the common approach is based on the concept of selective metal substitution. In this study, we propose an alternative approach, which is based on the formation of composites between individual phases having distinctions in the electric and thermal conductivities. Through rational manipulation of particle sizes and interfacial effects, it is possible to increase the power factor together with a reduction of the thermal conductivity, thus yielding the enhancement of the thermoelectric activity of composites in comparison with the individual constituents. Supporting this assumption, it has recently been found that hierarchical mesoscopic oxide composites of Ca_3_Co_4_O_9_ and La_0.8_Sr_0.2_CoO_3_ display enhanced thermopower [12]. However, the experimental observations of interfacial effects still remain elusive. There are several analytical techniques capable to probe the interfacial effects in composites; a common approach for their examination being absent. In this context, the synchrotron radiation-based X-ray photoemission electron microscopy (X-PEEM) experiments, combined with local X-ray absorption spectroscopy (XAS), are suitable for probing element specific properties of surfaces, interfaces, thin films, and nanomaterials [13]. In addition, the magnetic properties can provide indirect information on interfacial effects.

The aim of this study is to gather insight into the thermoelectric properties of multiphase composites formed between well-defined thermoelectric Co-based oxides: Ni, Fe co-substituted perovskite, LaCo_0.8_Ni_0.1_Fe_0.1_O_3_, and misfit layered Ca_3_Co_4_O_9_. Although the perovskite has lower thermal conductivity, the higher electrical conductivity is established for the misfit layered oxide [8,14]. The surface chemical and electrical properties of composites are probed with the means of SEM, PEEM/XAS, and X-ray photoemission spectroscopy (XPS) tools, as well as the magnetic susceptibility measurements. The thermoelectric power of the multiphase composites is evaluated by the dimensionless figure of merit ZT, calculated from the independently measured electrical resistivity (ρ), Seebeck coefficient (S), and thermal conductivity (λ).

## 2. Materials and Methods

### 2.1. Materials

Powders of Ca_3_Co_4_O_9_ were obtained by a Pechini-type reaction using citric acid (CA) and ethylene glycol (EG). The ratio between the elements in the solution was Ca:Co:CA:EG = 1:1:10:40. The solution was continuously heated at 90 °C in order to remove the excess of water and to intensify the polyesterification. The solid residue was decomposed at 400 °C for 3 h in air. The homogeneous powder was pelleted and heated at 800 °C for 15 h in air, followed by annealing at the same temperature for 5 h in an oxygen atmosphere. Perovskite oxides LaCo_0.8_Ni_0.1_Fe_0.1_O_3_ were also obtained by Pechini-type reactions. The details are given elsewhere [8,15].

**Figure 1:**
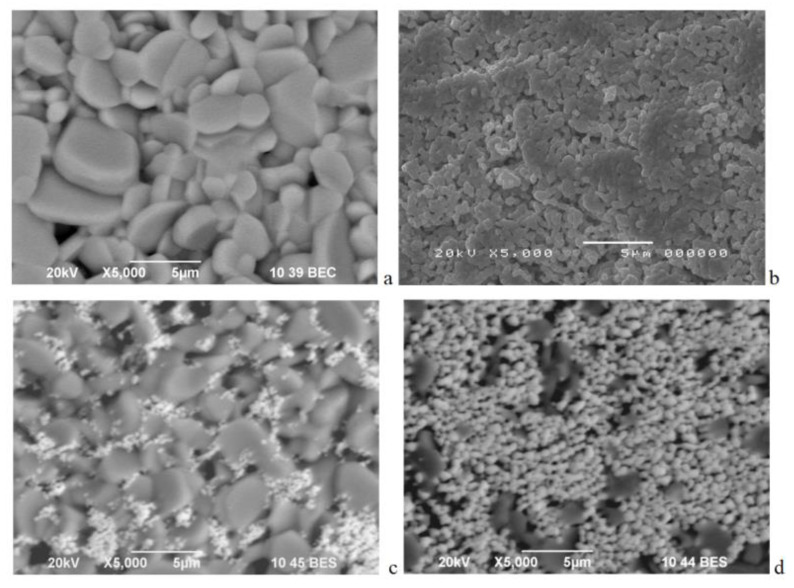
SEM images of Ca_3_Co_4_O_9_ (**a**), LaCo_0.8_Ni_0.1_Fe_0.1_O_3_ (**b**), LCO20Ca80 (**c**), and LCO80Ca20 (**d**).

The composites between LaCo_0.8_Ni_0.1_Fe_0.1_O_3_ and Ca_3_Co_4_O_9_ were prepared by mechanical mixing, followed by pelleting and annealing at 900 °C for 40 h in air. The ratio between LaCo_0.8_Ni_0.1_Fe_0.1_O_3_ and Ca_3_Co_4_O_9_ was 20-to-80 wt.% and 80-to-20 wt.%, for the composites further denoted as LCO20Ca80 and LCO80Ca20, respectively. These ratios were selected in order to achieve good packing between different particles (see Figure 1a,b), which was essential for improvement of the thermoelectric properties.

### 2.2. Characterization

X-ray structural analysis was performed on a Bruker Advance 8 diffractometer (Karlsruhe, Germany) with Cu Kα radiation. SEM images of pellets coated with a gold were carried out on a JEOL JSM 6390 scanning electron microscope (EDS, Oxford INCA Energy 350) in a regime of secondary electron image (SEI). The synchrotron X-PEEM experiments were performed at the National Synchrotron Radiation Centre SOLARIS at the soft X-ray bending magnet PEEM/XAS beamline [16], using the PEEM III microscope with the energy analyzer (ELMITEC Elektronenmikroskopie GmbH, Clausthal-Zellerfeld, Germany) with the energy analyzer. The surface analysis was accomplished by X-ray photoelectron spectroscopy and the measurements were carried out using an AXIS Supra electron spectrometer (Kratos Analytical Ltd., Manchester, UK), using Al Kα radiation with photon energy of 1486.6 eV. The energy calibration was performed by normalizing the C1s line of adsorbed adventitious hydrocarbons to 284.8 eV. The binding energies (BE) were determined with an accuracy of 0.1 eV, and the deconvolution of the peaks were performed using the commercial data-processing software ESCApeTM from Kratos Analytical Ltd. (Manchester, UK).

Electrical resistivity was measured by the four-probe method, with a source-measure unit Keithley 237. The samples were cooled down to liquid helium temperatures with a liquid helium free Advanced Research Systems closed cycle cryocooler. Carrier density (CD) and carrier mobility (CM) were determined by a MMR’s Variable Temperature Hall System (K2500-5SLP-SP, Mountain View, CA, USA) using the Van der Pauw method. The bench top permanent magnet (5 T) was used. Thermal conductivity was evaluated at room temperature on a C-Therm TCi Analyzer (Fredericton, NB, Canada). The magnetic susceptibility measurements were performed on a SQUID magnetometer (Quantum Design MPMSXL7). The Seebeck coefficient was measured using the Harman method. All measurements were performed on pellets having porosity between 20% and 25%. The pellets porosity was calculated by the Archimedes method.

## 3. Results

### 3.1. Multiphase Composites between Co-Based Perovskites and Layered Oxides

The annealing at 900 °C of the mechanical mixtures (i.e., LCO20Ca80 and LCO80Ca20) proceeded with the keeping of the structures and their individual components (Figure S1). The lattice parameters of both components LaCo_0.8_Ni_0.1_Fe_0.1_O_3_ and Ca_3_Co_4_O_9_ remained intact in the composites (Table S1), thus indicating a lack of chemical interaction between them.

The individual components LaCo_0.8_Ni_0.1_Fe_0.1_O_3_ and Ca_3_Co_4_O_9_ exhibited different morphology, which determined their sintering behaviour in composites (Figure 1). For the perovskite (Figure 1b), small round particles with uniform dimensions around 0.3–0.5 μm were formed, while for the layered oxide (Figure 1a), there were well faceted big particles with a mean size of around 2–5 μm. In the composite richer on layered oxide (i.e., LCO20Ca80), the bigger particles were densely packed so that the smaller perovskite particles filled the voids between them, while in the composite richer on perovskite (i.e., LCO80Ca20) there were close contacts between particles, since the small particles covered the big particles. In addition, the particle size distribution seemed narrower for the composite LCO20Ca80 than that of Ca_3_Co_4_O_9_, but the dominant particles were between 2 and 3 µm for both samples (Figure S2). This revealed that the small addition of LaCo_0.8_Ni_0.1_Fe_0.1_O_3_ constrained the particle growth of Ca_3_Co_4_O_9_, resulting in a closer distribution and a better particle packing.

**Figure 2:**
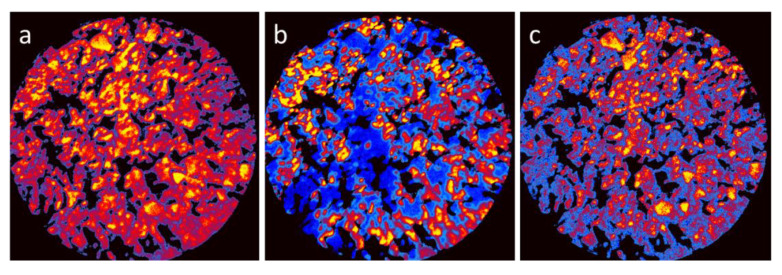
X-PEEM elemental maps of Co (**a**), La (**b**), and Ca (**c**) for the LCO20Ca80 composite, presented in the thermal palette. The black areas indicated the presence of voids, where the intensity was arbitrary set to zero. The field of view is 75 μm.

To correlate the surface topography with the spatially resolved chemical properties of the composites, the X-PEEM was employed. PEEM imaging best performed for flat and smooth surfaces, because roughness, which was naturally occurring for the present composite samples, deteriorated the image resolution [13]. Nevertheless, based on XAS surveys (details were presented in Supplementary Materials, Figure S3) it was possible to acquire images of the reasonable quality for the LCO20Ca80 composite, as shown in Figure 2. The presented images corresponded to absorption edges of the characteristic elements: Co L3-edge at 782 eV, Ca L2-edge at 352 eV, and La M5 edge at 836 eV, and they were obtained by normalizing the edge-images to images captured at energies 10 eV below the corresponding edges. In this way the chemical contrast was enhanced by reducing the topographic effects, and the images, presented in the thermal palette, could be regarded as elemental maps.

From the comparison of the X-PEEM elemental maps, separation of the perovskite and layered oxide phases was obvious. Whereas cobalt, present in both compounds, was rather evenly distributed over the entire surface, the areas rich in calcium were poor in lanthanum, and vice versa. Moreover, the distribution of Ca showed characteristic concentration in several micrometers grains, characteristic for Ca_3_Co_4_O_9_, and the distribution of La reflected rather the fine-grain morphology of perovskite. Alternatively, notwithstanding a poorer spatial resolution, the chemical maps indicated less distinct differentiation of the perovskite and layered oxide areas compared to the SEM images, which suggested diffused interfaces at the grain boundaries. This once again supported a development of intimate contacts between particles composed of perovskite and layered oxide.

**Figure 3:**
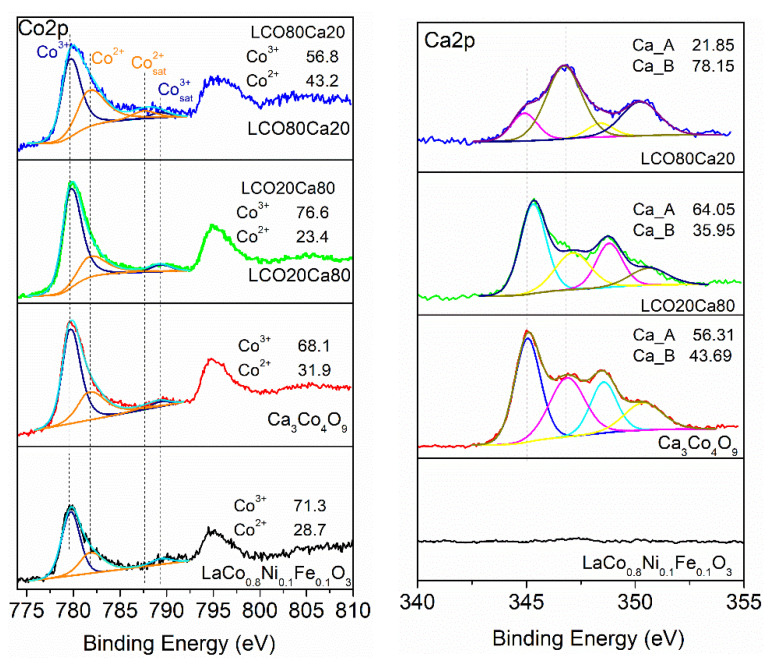
XPS spectra in the Co 2p (**left**) and Ca 2p (**right**) region for the perovskite and the layered oxide, as well as the composites between them.

In addition, the surface composition was monitored by XPS. The corresponding spectra in the energy range of La 3d, Ca 2p, and Co 2p, and the O 1s were collected on Figure 3. For the perovskite LCO, and the composites LCO20Ca80 and LCO80Ca20, the La 3d core level spectra displayed the well separated spin-orbit components (3d_3/2_ and 3d_5/2_) and each of them was composed of multiplet splitting. The binding energy for La 3d_5/2_ was around 834 eV, with energy differences between the 3d_3/2_ and the 3d_5/2_ levels of 17 eV, which corresponded to La^3+^ ions. Comparing perovskite LCO and composites LCO20Ca80 and LCO80Ca20, it appeared that the multiplet structure was well resolved for all samples, which indicated a close local structure for La in them.

The Ca 2p spectra were divided into two spin-orbit components, Ca 2p_3/2_ and Ca 2p_1/2_, and each spin-orbit component consisted of two overlapped peaks (Table 1). The binding energy at 345.7 eV for the first peak permitted to attribute it to calcium in the insulating Ca_2_CoO_3_ layer, [17] while the second peak at 346.8 eV came, most probably, from calcium in the carbonate impurity species [18]. The amount of the carbonate impurities was highest for the composite richer on the perovskite (i.e., LCO80Ca20).

The Co 2p_3/2_ spectra consisted at least of two overlapped peaks. Based on the satellite structure, these two peaks could be assigned to cobalt ions adopting high and low oxidation state of +3 and +2, respectively (Table 1). Although highly oxidized cobalt ions were stabilized in both the perovskite LCO and the CoO_2_ layer of Ca_3_Co_4_O_9_, the Co^2+^ ions resided mainly the Ca_2_CoO_3_ layer of Ca_3_Co_4_O_9_. [19,20] Therefore, the appearance of Co^2+^ in the perovskite LCO, as well as in the composites, could be related with their reducibility. Using strong reducing agents like H_2_, it had been found that Co^3+^ was reduced to Co^2+^ above 300 °C, without transforming the perovskite structure [21]. Supporting the suggestion on possible reduction of surface cobalt ions, Table 1 gave the relative amounts of Co^3+^ and Co^2+^ ions. As shown, the relative amounts of cobalt ions vary in a nonsystematic way over the sample compositions.

The O 1s spectra could be deconvoluted into two broad peaks due to the lattice oxygen (at around 528.6–529.0 eV) and impurity carbonate and/or hydroxide species (531.5 eV). This was in line with the Ca 2p spectra, where impurity carbonate species were also observed.

**Figure 4:**
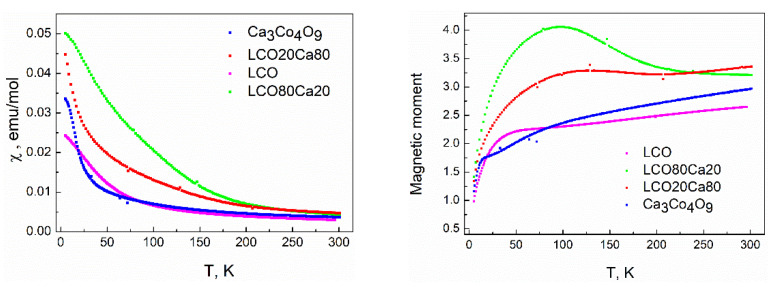
Temperature dependence of the magnetic susceptibility (**left**) and calculated effective magnetic moment (**right**) for the individual components and the composites between them.

The magnetic properties had been considered as the most sensitive indirect tools to assess the interfacial effects in materials. Figure 4 showed the temperature dependence of the magnetic susceptibility and the calculated magnetic moment for the individual components and the composites between them. In the case of the layered oxide Ca_3_Co_4_O_9_, a magnetic transition at 20 K was clearly visible, and it could be associated with the ferromagnetic transition previously established for powdered Ca_3_Co_4_O_9_ [3,22]. Between 180 and 300 K, the Curie-Weiss law was obeyed. The experimentally calculated values of the magnetic moment and the Weiss constant were 2.13 and −182 K, respectively. The value of the magnetic moment was close to that value (i.e., 1.96) calculated if we assumed that Co^3+^ are in mixed spin states (i.e., 0.33 part of Co^3+^ was in high spin and the resting one was in a low spin), while Fe^3+^ and Ni^3+^ ions adopted high and low spin states (i.e., 5.92 and 1.73), respectively. Below 50 K, there was an irrupted decrease in the magnetic moment (Figure 4). This reflected the temperature-induced transition of Co^3+^ from a high spin to a low spin state. The same transition from high- to low-spin state had already been established for unsubstituted LaCoO_3_ perovskite (i.e., at around 100 K), [23,24].

The formation of the composites between perovskite and layered oxides led to a dramatic change in the temperature dependence of the magnetic susceptibility (Figure 4). In contrast to the individual component Ca_3_Co_4_O_9_, the ferromagnetic transition at 20 K disappeared at the composite richer on the layered oxide. In addition, the magnetic moment increased on heating from low temperatures; reaching a maximum between 75 and 100 K, after which there was a decrease. The same temperature evolution was observed at the composite richer on the perovskite: lack of the ferromagnetic transition at 20 K and a broad temperature range where the magnetic moment had the highest values. These data revealed the different magnetic properties of the composites compared to that of the individual components, which, on its turn, could be related with the diffused interfaces at the grain boundaries between perovskite and layered oxide (as was established by X-PEEM experiments, Figure 2).

### 3.2. Thermoelectric Properties of Multiphase Composites

**Figure 5:**
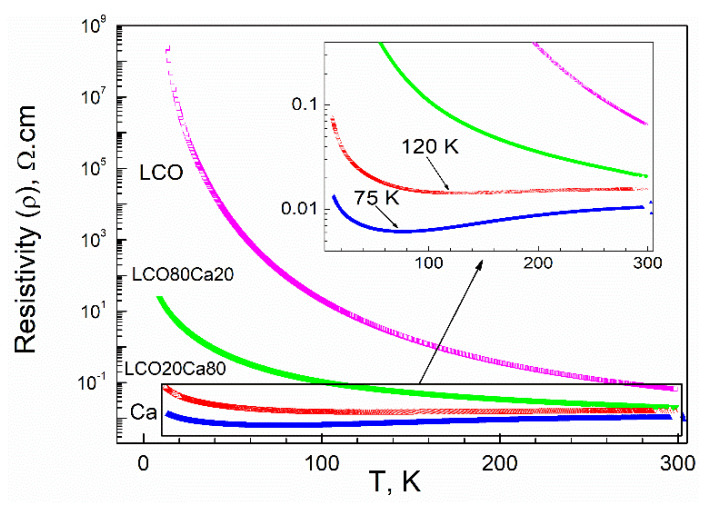
Temperature dependence of the electrical resistivity for the individual components (perovskite, LCO, and layered oxide, Ca) and the composites between them (LCO80Ca20 and LCO20Ca80).

In agreement with the magnetic properties, the electric properties of the composites further deviated from that of the individual components (Figure 5). The perovskite LCO was a semiconducting material and exhibited higher electrical resistivity than that of the layered oxide in the temperature range of 5–300 K [25]. The resistivity of the layered oxide Ca_3_Co_4_O_9_ decreased on cooling from 300 K, reaching a broad minimum around 75 K, followed by an increase below 75 K. This behavior of Ca_3_Co_4_O_9_ had been explained by a metal–insulator transition occurring at about 80 K and, below 80 K, a semiconducting-like behavior was realized [3]. For the composite richer on the layered oxide (LCO20Ca80), the broad minimum in the electrical resistivity was shifted from 75 K up to 120 K and, below this temperature, a sharp increase in the electrical resistivity was observed. For the composite richer on the perovskite, the electrical resistivity increased without showing any peculiarities as in the case of the perovskite LCO. It is of importance that, in the whole temperature range from 5 to 300 K, both composites LCO20Ca80 and LCO80Ca20 were characterized with the electric resistivity that was an intermediate between that of the perovskite and the layered oxide. Thus, the electric properties provided further evidence that interface between perovskite and the layered oxide affected the transport properties of composites.

The difference in the electric properties of individual components and the composites can be explained considering the mobility and density of the charge carriers (Table 2). The comparison of all data disclosed that the carrier mobility was a main parameter determining the difference in the electric resistivity. The higher the carrier mobility, the lower the electrical resistivity. In contrast to the carrier mobility, the carrier density varied around 10 ^18^ cm^−3^ and showed a tendency to decrease when going from perovskite to the layered oxide. Thus, at 298 K, the layered oxide had a carrier mobility two orders higher than that of the perovskite and it became more conductive. The improved carrier mobility could be related with the mixed oxidation states of cobalt ions stabilized in the structures of the individual components: the misfit layered structure appeared to be more flexible to accommodate cobalt ions in mixed oxidation states (Co^2+^, Co^3+^, and/or Co^4+^), while the octahedral perovskite sites were mainly occupied by highly oxidized cobalt ions. It is noteworthy to mention that the complete oxidation of Co^3+^ to Co^2+^ in LaCoO_3_ proceeded with a transformation of the perovskite into a Brown-Millerite type of structure [21,26,27]. For the composites, the carrier mobility increased with increasing the amount of the layered oxide, which resulted in enhanced electric conductivity.

For all samples, the Seebeck coefficient (S) had a positive sign, which meant that the holes were predominant mobile charge carriers. In addition, the Seebeck coefficient of the perovskite was slightly higher than that of the layered oxide (Table 2). Notably, the composites display the Seebeck coefficient that was comparable with that of the predominant component. The small variation in the Seebeck coefficient correlated well with the carrier density determined for the individual components and composites (Table 2). Supporting this correlation, it had been experimentally demonstrated that, in the high-temperature limit, the magnitude of Seebeck coefficient for cobaltates was satisfactory predicted, considering the carrier density, spin, and orbital degrees of freedom (i.e., well-known formula of Heikes and Koshibae) [28,29]. Since all composites contained Co ions with corresponding spin and orbital degrees of freedom, the Seebeck coefficient was mainly determined by the carrier density (Table 2).

Based on the Seebeck coefficient and electric resistivity data, the power factor was calculated PF = S^2^/ρ (Table 2). As shown, the PF of the layered oxide was two times higher than that of the perovskite. This was a result of the huge electric conductivity of the layered oxide (Table 2). It was interesting that the addition of the layered oxide to the perovskite composite led to an enormous enhancement of the PF, so that its value became higher than those of the individual components (Table 2). After the addition of the perovskite to the layered oxide, the PF of the composite slightly decreased (Table 2). All of these data underlined the important roles of the electric conductivity of the composites, which, were governed by the diffuse interface between individual components.

The thermal conductivity was the next parameter that governed the thermoelectric efficiency of oxides (Table 2). The perovskite displayed significantly lower thermal conductivity in comparison with that of the layered oxide. Given that the thermal conductivity was a function of the conductive carriers and the phonon scattering, the lower thermal conductivity of the perovskite (having slightly higher charge carriers than the layered oxide) suggested that the lattice contribution to the thermal conductivity was more important. The thermal conductivities of the composites were intermediate between the thermal conductivities of the individual components: going from the composite richer on the perovskite to the composite richer on the layered oxide, the thermal conductivity increased (Table 2). The smooth variation in the thermal conductivity of the composites implied a major role of the lattice in the phonon scattering instead of the interfacial effects. It appeared that the perovskite structure more effectively reduced the heat transport in the cobaltates than the misfit layered structure.

Combining the data on the Seebeck coefficient, electric, and thermal conductivity, the figure-of-merit was calculated (Table 2). Thanks to the lower thermal conductivity, the perovskite had higher thermoelectric activity than the misfit layered oxide. Further improvement in the thermoelectric efficiency was achieved at the composite, in which small amounts of the layered oxide was added to the perovskite. In contrast, the composite richer on the layered oxide exhibited a thermoelectric activity, which was intermediate between two end components (perovskite and layered oxide). Irrespective of this, the important finding here was that the thermoelectric activity of the composites outperformed that of the individual components. This could be related with the effects of the composite interfaces on the electric and thermal conductivity. It is worth mentioning that, at room temperature, the thermoelectric activity of the composites outperformed the previously reported layered cobalt oxide epitaxial films having the highest figure-of-merit (i.e., ZT = 0.11) [30].

## 4. Conclusions

The composites between perovskite, LaCo_0.8_Ni_0.1_Fe_0.1_O_3_, and misfit layered oxide, Ca_3_Co_4_O_9_, phases were formed after annealing at 900 °C in air atmosphere. In the composites, the structure of the individual components remained intact. The morphology of the composites was based on an intimate contact between smaller perovskite particles (with sizes around 0.3–0.5 µm) and bigger particles composed of the layered oxide (around 2–5 µm). The sintering of particles depended on the relative amount of the individual components. At the composite richer on the layered oxide, the larger particles of Ca_3_Co_4_O_9_, were connected with each other and the voids between them were filled with smaller perovskite particles. At the composite richer on the layered oxide, the perovskite particles covered the particles composed of layered oxide. The diffuse interfaces between perovskite and layered oxide caused a shift of the ferromagnetic transition of the layered oxide (i.e., at 20 K) to higher temperatures, as well as affected the transition of Co ions from low- to high-spin state.

The power factor of the layered oxide was higher than that of the perovskite, but the lower thermal conductivity of the perovskite turned the order opposite. The magnitudes of electric and thermal conductivities of the composites depended more significantly on the interfaces than the Seebeck coefficient values. As a result, the highest thermoelectric activity was observed at the composite richer on the perovskite (i.e., ZT = 0.34 at 298 K). This was a consequence of the low thermal conductivity and high electric conductivity, while the Seebeck coefficient was comparable with that of the individual perovskite component. The composite richer on the layered oxide was characterized with slightly higher thermoelectric activity than that of Ca_3_Co_4_O_9_. The formation of composites between phases, each of them having low thermal conductivity and high electric conductivity, was an effective way to modify the thermoelectric activity of the cobaltates.

## Figures and Tables

**Table 1:** Binding energies and relative amount of Co^3+^, Co^2+^, Ca^2+^ (A), and Ca^2+^ (B) for the perovskite and layered oxides and composites between them.

Samples	Co 2p		Binding Energy, eV				Ca 2p		Binding Energy, eV			
	Co^3+^	Co^2+^	Co^3+^	Co^2+^	Co^2+^ sat	Co^3+^ sat	Ca (A)	Ca (B)	Ca^2+^ (A) 2p_3/2_	Ca^2+^ (A) 2p_3/2_	Ca^2+^ (B) 2p_1/2_	Ca^2+^ (B) 2p_1/2_
LCO	71.3	28.7	779.6	781.9	787.6	789.3	-	-	-	-	-	-
LCO80Ca20	56.8	43.2					21.9	78.1	345.0	346.8	348.8	350.5
LCO20Ca80	76.6	23.4					64.0	36.0	345.0	346.8	348.8	350.5
Ca_3_Co_4_O_9_	68.1	31.9					56.3	43.7	345.0	346.8	348.8	350.5

**Table 2:** Electrical resistivity (ρ), carrier mobility (CM), and carrier density (CD) at 298 K for individual components (LaCo_0.8_Ni_0.1_Fe_0.1_O_3_, LCO, and Ca_3_Co_4_O_9_, Ca) and composites between them (LCO80Ca20 and LCO20Ca80).

Samples	ρ (Ω·cm) at 298 K	CM (cm^2^/Vs) at 298 K	CD (cm^−3^) at 298 K	S, mV/K	PF PF = S2/ρ, μW/(K^2^·cm)	λ, W/(m·K) ±0.04	Figure of Merit S^2^T/(ρ·λ) T = 298K
LCO	0.065	2	3.0 × 10^18^	234	0.84	0.157	0.16
LCO80Ca20	0.021	11	4.2 × 10^18^	208	2.06	0.179	0.34
LCO20Ca80	0.012	49	1.1 × 10^18^	137	1.56	0.467	0.10
Ca_3_Co_4_O_9_	0.010	1050	0.7 × 10^18^	130	1.69	0.620	0.08

## Data Availability

Not applicable.

## Supplementary Materials

The following are available online at https://www.mdpi.com/article/10.3390/ma14227019/s1, Figure S1: XRD patterns of individual components LaCo_0.8_Ni_0.1_Fe_0.1_O_3_ and Ca_3_Co_4_O_9_ and composites between them LCO80Ca20 and LCO20Ca80. Bragg reflections for the perovskite, [Ca_2_CoO_3_] and [CoO_2_] blocks, and [Ca_3_Co_4_O_9_] compositions are given. The detailed structural analysis of rhombohedrally distorted perovskite is given elsewhere [1]. Table S1: Lattice parameters (*a*, *c*, *V*) for LaCo_0.8_Ni_0.1_Fe_0.1_O_3_, LCO80Ca20, LCO20Ca80 and Ca_3_Co_4_O_9_ Figure S2. Particle size distribution calculated from SEM images of LCO20Ca80 and Ca_3_Co_4_O_9_; Figure S3. XAS spectra for the LCO20Ca80 composite taken from the areas marked with corresponding squares in the accompanying image. The image is taken at an energy close to the Co-L3 edge, the field of view is 75 μm. With the top spectrum, integrated over the big grey square, adsorption edges are identified. Small features at half-energies of the Co and La edges are due to the second order diffraction at monochromator. The red spectrum, with dominating La-intensity, shows that the fine-grain structure areas are perovskite-rich. The green spectrum, with considerable contribution from dark low intensity area, identified as a void, indicates domination of Ca and Co rich layered oxide.

## References

[1] Feng Y., Jiang X., Ghafari E., Kucukgok B., Zhang C., Ferguson I., Lu N. (2018). Metal oxides for thermoelectric power generation and beyond. Adv. Compos. Hybrid Mater..

[2] Singh S., Pandey S.K. (2017). Understanding the thermoelectric properties of LaCoO_3_ compound. Philos. Mag..

[3] Fergus J.W. (2012). Oxide materials for high temperature thermoelectric energy conversion. J. Eur. Ceram. Soc..

[4] Herve P., Ngamou T., Bahlawane N. (2010). Influence of the arrangement of the octahedrally coordinated trivalent cobalt cations on the electrical charge transport and surface reactivity. Chem. Mater..

[5] Hébert S., Flahaut D., Martin C., Lemonnier S., Noudem J., Goupil C., Maignan A., Hejtmanek J. (2007). Thermoelectric properties of perovskites: Sign change of the Seebeck coefficient and high temperature properties. J. Prog. Solid State Chem..

[6] Dho J., Hur N.H. (2006). Magnetic and transport properties of lanthanum perovskites with B-site half doping. Solid State Commun..

[7] Luo X., Xing W., Li Z., Wu G., Chen X. (2007). Impact of the Fe doping on magnetism in perovskite cobaltites. Phys. Rev. B.

[8] Vulchev V., Vassilev L., Harizanova S., Khristov M., Zhecheva E., Stoyanova R. (2012). Improving of the thermoelectric efficiency of LaCoO_3_ by double substitution with Nickel and Iron. J. Phys. Chem. C.

[9] Harizanova S., Zhecheva E., Valchev V., Khristov M., Stoyanova R. (2015). Improving the thermoelectric efficiency of Co based ceramics. Mater. Today Proc..

[10] Klie R.F., Qiao Q., Paulauskas T., Gulec A., Rebola A., Öğüt S., Gupta A. (2012). Observations of Co^4+^ in a higher spin state and the increase in the Seebeck coefficient of thermoelectric Ca_3_Co_4_O_9_. Phys. Rev. Lett..

[11] Huang Y., Zhao B., Lin S., Sun Y. (2015). Enhanced Thermoelectric Performance Induced by Misplaced Substitution in Layered Ca_3_Co_4_O_9_. J. Phys. Chem. C.

[12] Butt S., Xu W., Farooq M.U., Ren G.-K., Mohmed F., Lin Y., Nan C.W. (2015). Enhancement of thermoelectric performance in hierarchical mesoscopic oxide composites of Ca_3_Co_4_O_9_ and La_0.8_Sr_0.2_CoO_3_. J. Am. Ceram. Soc..

[13] Feng J., Hawkes P.W., Spence J.C.H. (2007). Scholl, Photoemission Electron Microscopy (PEEM). Science of Microscopy.

[14] Miyazawa K., Amaral F., Kovalevsky A.V., Graça M.P.F. (2016). Hybrid microwave processing of Ca_3_Co_4_O_9_ thermoelectrics. Ceram. Int..

[15] Harizanova S., Zhecheva E., Valchev V., Markov P., Khristov M., Stoyanova R. (2016). Effect of multiple metal substitutions for A- and B- perovskite sites on the thermoelectric properties of LaCoO_3_. Inter. J. Sci. Res. Sci. Techn..

[16] Zając M., Giela T., Freindl K., Kollbek K., Korecki J., Madej E., Pitala K., Kozioł-Rachwał A., Sikora M., Spiridis N. (2021). The first experimental results from the 04BM (PEEM/XAS) beamline at Solaris. Nucl. Instrum. Methods Phys. Res. Sect. B Beam Interact. Mater. Atoms.

[17] Dupin J.C., Gonbeau D., Vinatier P., Levasseur A. (2000). Systematic XPS studies of metal oxides, hydroxides and peroxides. Phys. Chem. Chem. Phys..

[18] Sosulnikov M.I., Teterin Y.A. (1992). X-ray photoelectron studies of Ca, Sr and Ba and their oxides and carbonates. J. Electron. Spectrosc. Relat. Phenom..

[19] Daheron L., Dedryvere R., Martinez H., Menetrier M., Denage C., Delmas C., Gonbeau D. (2008). Electron transfer mechanisms upon lithium deintercalation from LiCoO_2_ to CoO_2_ investigated by XPS. Chem. Mater..

[20] Liu H., Lin G.C., Ding X.D., Zhang J.X. (2013). Mechanical relaxation in thermoelectric oxide Ca_3−x_Sr_x_Co_4_O_9+δ_ (x = 0, 0.25, 0.5, 1.0) associated with oxygen vacancies. J. Solid State Chem..

[21] Ivanova S., Senyshyn A., Zhecheva E., Tenchev K., Nikolov V., Stoyanova R., Fuess H. (2009). Effect of the synthesis route on the microstructure and the reducibility of LaCoO_3_. J. Alloys Compd..

[22] Sugiyama J., Itahara H., Tani T., Brewer J.H., Ansaldo E.J. (2002). Magnetism of layered cobalt oxides investigated by muon spin rotation and relaxation. Phys. Rev. B.

[23] Zhou S., He L., Zhao S., Guo Y., Zhao J., Shi L. (2009). Size-dependent structural and magnetic properties of LaCoO_3_ nanoparticles. J. Phys. Chem. C.

[24] Plakhty V.P., Brown P.J., Grenier B., Shiryaev S.V., Barilo S.N., Gavrilov S.V., Ressouche E. (2006). Thermal excitation of the Co^3+^ triplet spin-state in LaCoO_3_ determined by polarized neutron diffraction. J. Phys. Condens. Matter..

[25] Jirák Z., Hejtmánek J., Knížek K., Veverka M. (2008). Electrical resistivity and thermopower measurements of the hole- and electron-doped cobaltites LnCoO_3_. Phisical Rev. B.

[26] Huang H., Zhang J., Zhang H., Han F., Chen X., Song J., Zhang J., Qi S., Chen Y.-H., Cai J. (2020). Topotactic transition between perovskite and brownmillerite phases for epitaxial LaCoO_3−*δ*_ films and effects thus resulted. J. Phys. D Appl. Phys..

[27] Charello G.L., Grunwald J.-D., Ferri D., Krumeich F., Oliva C., Forni L., Baiker A. (2007). Flame-synthesized LaCoO_3_-supported Pd: 1. Structure, thermal stability and reducibility. J. Catal..

[28] Chaikin P.M., Beni G. (1976). Thermopower in the correlated hopping regime. Phys. Rev. B.

[29] Koshibae W., Tsutsui K., Maekawa S. (2000). Thermopower in cobalt oxides. Phys. Rev. B.

[30] Takashima Y., Zhang Y., Wei J., Feng B., Ikuhara Y., Jun Cho H., Ohta H. (2021). Layered cobalt oxide epitaxial films exhibiting thermoelectric ZT = 0.11 at room temperature. J. Mater. Chem. A.

