# Supplementary material for: Composites between Perovskite and Layered Co-Based Oxides for Modification of the Thermoelectric Efficiency
Source: Materials (Basel). 2021 Nov 19;14(22):7019. doi: 10.3390/ma14227019 (PMC8624505; doi:10.3390/ma14227019)
Supplement: Supplementary file 1 [file materials-14-07019-s001.zip › materials-1448346-supplementary.pdf]

materials

MDPI

Supplementary Materials: Composites between Perovskite and Layered Co-based Oxides for Modification of the Thermoelectric Efficiency

Sonya Harizanova [1], Eric Faulques [2], Benoit Corraze [2], Christophe Payen [2], Marcin Zając [3], Dorota Wilgocka-Ślęzak [4], Józef Korecki [3,4], Genoveva Atanasova [1] and Radostina Stoyanova [1,*]

1 Institute of General and Inorganic Chemistry, Bulgarian Academy of Sciences, 1113 Sofia, Bulgaria; sonya@svr.igic.bas.bg (S. H); genoveva@svr.igic.bas.bg (G.A.); radstoy@svr.igic.bas.bg (R.S.)
2 University of Nantes, CNRS, Institut des Matériaux Jean Rouxel, IMN, F-44000 Nantes, France; Eric.Faulques@cnrs-imn.fr (E.F); Benoit.Corraze@cnrs-imn.fr (B.C.); christophe.payen@cnrs-imn.fr (C.P.)
3 National Synchrotron Radiation Centre Solaris, 30-392 Kraków, Poland; mar.zajac@uj.edu.pl (M.Z.), jozef.korecki@ikifp.edu.pl (J.K.)
4 Jerzy Haber Institute of Catalysis and Surface Chemistry, Polish Academy of Sciences, 30-239 Kraków, Poland; dorota.wilgocka-slezak@ikifp.edu.pl (D.-W.S.)
* Correspondence: radstoy@svr.igic.bas.bg; Tel.: +35929793915.

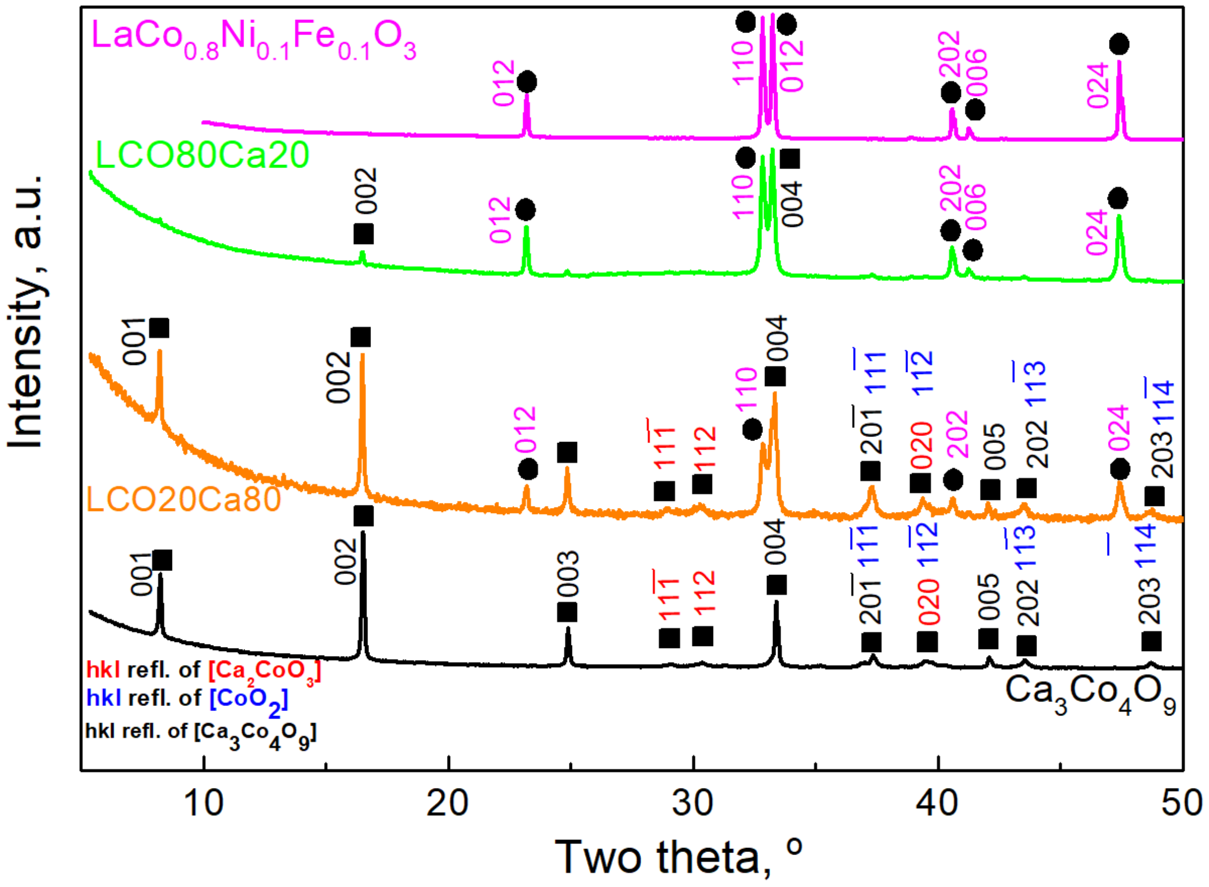

Figure S1. XRD patterns of individual components $LaCo_{0.8}Ni_{0.1}Fe_{0.1}O_3$ and $Ca_3Co_4O_9$ and composites between them LCO80Ca20 and LCO20Ca80. Bragg reflections for the perovskite, [Ca_2CoO_3] and [CoO_2] blocks, and [$Ca_3Co_4O_9$] compositions are given. The indexation of XRD patterns is based on the ICSD collection codes for $LaCoO_3$ (17668) and $Ca_2CoO_3(CoO_2)_{1.62}$ (55458). The detailed structural analysis of rhombohedrally distorted perovskite is given elsewhere [1].

Table S1. Lattice parameters (*a*, *c*, *V*) for $LaCo_{0.8}Ni_{0.1}Fe_{0.1}O_3$, LCO80Ca20, LCO20Ca80 and $Ca_3Co_4O_9$:

Samples	Unit cells		
	a±0.0001, *Å*	*c*±0.0001, *Å*	*V*, *Å*^{3}
$LaCo_{0.8}Ni_{0.1}Fe_{0.1}O_3$	5.4532	13.1191	337.86
LCO80Ca20	..	..	..
LCO20Ca80	..	..	..

**Samples**	**Unit cells**						
	a±0.0001, *Å*	*b*$_1$±0.0001, *Å*	*c*±0.0001, *Å*	*b*$_2$±0.0001, *Å*	β	*V*$_1$, *Å*^{3}	*V*$_2$, *Å*^{3}
LCO80Ca20	4.8370	4.5618	10.8960	2.8081	97.7651	238.22	146.64
LCO20Ca80	4.8398	4.5660	10.8157	2.8020	98.2447	236.54	145.16
$Ca_3Co_4O_9$	4.8249	4.5687	10.8623	2.8096	98.3676	236.90	145.68

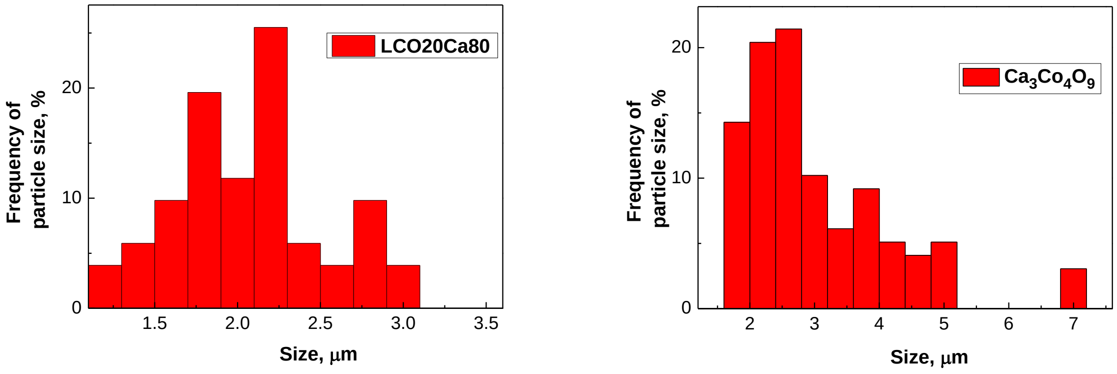

Figure S2. Particle size distribution calculated from SEM images of LCO20Ca80 and $Ca_3Co_4O_9$.

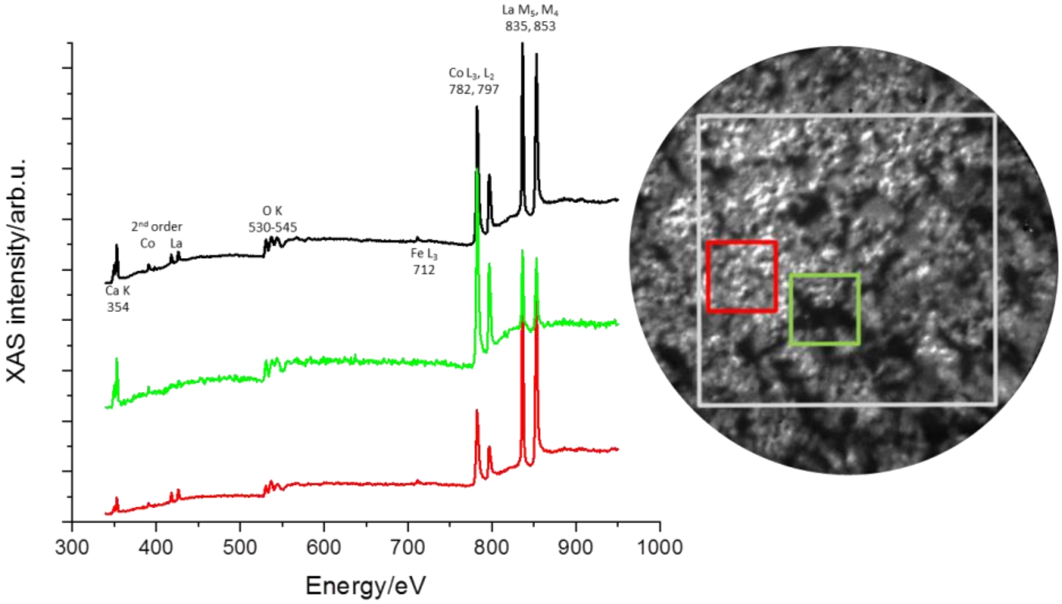

Figure S3. XAS spectra for the LCO20Ca80 composite taken from the areas marked with corresponding squares in the accompanying image. The image is taken at an energy close to the Co-L3 edge, the field of view is 75 µm. With the top spectrum, integrated over the big grey square, adsorption edges are identified. Small features at half-energies of the Co and La edges are due to the second order diffraction at monochromator. The red spectrum, with dominating La-intensity, shows that the fine-grain structure areas are perovskite-rich. The green spectrum, with considerable contribution from dark low intensity area, identified as a void, indicates domination of Ca and Co rich layered oxide.

© 2021 by the authors. Submitted for possible open access publication under the terms and conditions of the Creative Commons Attribution (CC BY) license (https://creativecommons.org/licenses/by/4.0/).
